# Protein Knowledge of Older Adults and Identification of Subgroups with Poor Knowledge

**DOI:** 10.3390/nu13031006

**Published:** 2021-03-20

**Authors:** Marjolein Visser, Yung Hung, Wim Verbeke

**Affiliations:** 1 Department of Health Sciences, Faculty of Science, Vrije Universiteit Amsterdam, De Boelelaan 1085, 1081 HV Amsterdam, The Netherlands; 2 Department of Agricultural Economics, Ghent University, Coupure Links 653, B-9000 Ghent, Belgium; Yung.Hung@UGent.be (Y.H.); wim.verbeke@ugent.be (W.V.)

**Keywords:** nutrition, aging, communication, consumer, information source, protein-energy malnutrition (PEM)

## Abstract

The aim was to investigate the protein knowledge of community-dwelling older adults. A survey was conducted among 1825 adults aged ≥65 years and living in Finland, Netherlands, Poland, Spain and United Kingdom in 2017. Protein knowledge was measured with nine objective knowledge statements provided only to participants who indicated to know what the nutrient "protein" is (64.7% of sample). Demographic, socioeconomic and health determinants of poor protein knowledge were investigated using multiple logistic regression analyses. The sample was 49.6% female and 87.0% reported no walking difficulties. Participants scored best on the true statement "You need protein in the diet for repairing bones and muscles" (89.3% correct), and worst on the false statement "One meal per day with a good protein source is sufficient" (25.4% correct). Median knowledge score was 5.0 (scale 0–9) and poor knowledge was present in 49.4% of the sample. Males (Odds Ratio 1.57), those unable to walk for 5 min (2.66), not always making their own food decision (1.36) and having lower income (1.44) were more likely to have poor knowledge. Large differences were observed across countries. In conclusion, poor protein knowledge is present in about half of community-dwelling older adults. Communication strategies should be tailored to target the identified risk groups with poor knowledge.

## 1. Introduction

Sufficient protein in the diet is necessary to maintain optimal health of older adults. In observational studies, a lower protein intake (less than 0.8 g per kilogram per day) in community-dwelling older adults has been associated with accelerated muscle mass loss [1] and decline in physical function [2]. Despite these elevated health risks associated with a lower protein intake, a substantial number of older adults do not meet the current protein intake recommendation. A recent study, using data from both national dietary surveys and large aging cohorts, indicated that 29.1% of community-dwelling older adults does not meet the recommendation of 0.8 g of protein per kilogram body weight per day [3]. When using higher cut-off values for the recommended protein intake, as advised previously by some expert groups [4,5] and applied by some countries, 54.3% had an intake below 1.0 g/kg/day, and 75.7% an intake below 1.2 g/kg/day. These results suggest that a suboptimal protein intake is highly prevalent in older adults and that protein intake behaviour needs to be better understood and improved for many older adults.

Older adults’ reasons to consume protein-rich foods include cognitive-based reasons, such as nutritional knowledge and health beliefs, but also product-based reasons (such as liking and appearance) and environment-based reasons (such as convenience, living status and spoilage) [6]. Furthermore, increasing protein knowledge through a nutrition information program specifically targeting protein consumption increased actual protein intake in older community-dwelling adults [7]. These studies suggest that protein knowledge in older adults is directly linked to protein intake. However, information on the level of objective knowledge about dietary protein of older adults is limited. Such information is crucial in order to develop effective communication guidelines aiming to increase protein intake. Furthermore, characteristics of older adults with lower protein knowledge need to be identified in order to target specific population groups.

Communication and information can lead to an improvement in dietary behaviour [8]. In order for communication strategies to reach older adults and be effective, communication routes that are already used by and familiar to older adults to obtain information are preferred. Moreover, the communication routes should be specific to the targeted subgroups. Therefore, commonly used information sources by older adults need to be identified in order to support communication guidelines for optimal protein intake strategies.

The objectives of this study were twofold. The first objective was to investigate the level of knowledge about dietary protein among European older adults, and the demographic, socioeconomic and health characteristics associated with poor knowledge. The second objective was to identify the most frequently used communication routes to obtain information on new food products by older adults with lower and higher protein knowledge. 

## 2. Methods

### 2.1. Data Collection

A cross-sectional online survey was conducted in five EU countries (Finland, the Netherlands, Poland, Spain and the United Kingdom) in June 2017 (*n* = 1825 in total and *n* = ±365 per country). The study sample included older adults who are 65 years or above and live independently with or without assistance. The five countries were selected to represent the large variation within Europe with regard to geographical location (north–south as well as east–west), cultural eating habits (e.g., with regard to the planning and composition of warm meals and the use of specific types of protein-rich foods), and the current national protein intake recommendation for older adults (0.8 versus 1.2 g of protein per kilogram body weight per day, in, e.g., Netherlands and Finland, respectively). The Belgian Ethics Committee of Ghent University Hospital granted ethics approval for the study in March 2017 (Reference No. B670201422567). Participants were recruited from the online access proprietary panel of a professional market research agency using probabilistic sampling. A nationally representative sample in terms of gender and region in each of the study countries was achieved by setting recruitment quota. The invitation and questionnaire administration were handled electronically by the market research agency. The questionnaire was developed in English, then reviewed by experts in the research consortium with experience and expertise in geriatric subjects for content, wording, and expected understanding of the participants. Then, it was translated into the respective national languages by a professional translation office and proofread by the native speakers who were affiliated with the research consortium. Prior to the actual field work, the questionnaire was pretested through survey field interviews in a sample of about 30 participants in each of the study countries for overall clarity of content and length of the survey. All data were collected and coded in a non-identifiable format and processed anonymously.

### 2.2. Questionnaire Content 

Participants first received a short description of the EC-funded, Horizon 2020 PROMISS project and the informed consent. Then, they were screened based on gender, age, country and current living condition before entering the survey. Other results of the survey were published previously [9,10]. 

Knowledge of dietary protein was measured by using a dichotomous question probing for perceived knowledge as a filter followed by a series of nine true and false statements regarding dietary protein probing for objective knowledge. The true/false statements covered three aspects of nutritional knowledge [11]: knowledge on the relationship between protein and health, knowledge on the protein content of foods, and knowledge of the recommended protein intake. In order to avoid bias from eventual random responding on the objective knowledge items, only participants who indicated "Yes" to the filter question "Do you know what dietary protein is?" were directed to the objective knowledge test based on nine statements. Participants had to answer one of the three options "True", "False" or "I do not know" to each of the statements. The full text of the statements is shown in the corresponding tables in the results section. An answer was considered correct when a true statement was considered true, or a false statement was considered false. The objective protein knowledge score based on the statements was obtained through aggregating the number of correct answers and could thus range from 0 to 9. A lower protein knowledge was defined as either (1) answering "No" to the filter question "Do you know what dietary protein is?" or (2) an objective protein knowledge score lower than 5 (median score).

After completing the protein knowledge section of the questionnaire, textual information was provided to explain all participants what protein is: ”Proteins are in every human body cell. Our bodies get proteins from the foods we eat to build and maintain bones, muscles and skin. Good sources of dietary proteins include meats and fish, dairy products, nuts, beans and certain grains. The amount of protein that you need depends on your age, gender, health and level of physical activity”. Hence, all participants were informed to the same level about what protein is before proceeding. Next, participants were asked "What do you think about the amount of protein in your daily diet?" which they could answer using a five-point-scale ranging from "Too much" to "Too little" or "I do not know". They were also asked whether they intend to change the amount of protein in their daily diet, with the response options "Yes, increase the amount", "Yes, decrease the amount", "No, remain the same" or "I do not know". Participants were also asked if they would increase the amount of protein in their daily diet if they were told to do so by a health professional, food industry, family or friends ("Yes", "No" and "I don’t know"). 

Frequency of information acquisition from different types of media and different information sources was assessed through a five-point frequency scale, ranging from "Never" to "Always". Participants received the question "How often do you get information about new food products from the following media and information sources?" modified based on Pieniak et al. [12] wherein 11 types of media, e.g., Television, and 8 types of information sources, e.g., Physicians/Dieticians, were provided. 

The survey ended with a series of questions used to assess the demographic, socioeconomic and health characteristics of participants. The individual items have been elaborated in Hung et al. [9]. 

### 2.3. Statistical Analysis

SPSS Statistics 25.0 (IBM SPSS, Armonk, NY, USA) was used to perform the statistical analyses. Differences in the percentage with a correct answer of each protein knowledge statement between those with lower and higher protein knowledge were tested using chi-square tests. Multiple logistic regression analysis was used to identify the demographic, socioeconomic and health characteristics that were associated with a lower protein knowledge. A lower knowledge about dietary protein was the dependent variable. The explanatory variables included demographic (i.e., gender, age, country of living, household composition (living alone or with others)), socioeconomic (education level, food shopping responsibility, own meal decision, food expenditure, household income, perceived financial situation), and health characteristics (diet status, ability to prepare own warm meals, ability to walk or move own wheelchair, and BMI calculated from self-reported body height (cm) and body weight (kg)). Participants who answered "Prefer not to say/Do not know" to the questions related to their household income or financial situation (*n* = 236), or answered "I never do it/I am using an electric wheelchair" to the questions about mobility for 5 min without resting were excluded from the multivariate analysis (*n* = 3). The assumptions for multiple logistic regression analysis were tested [13]. There was no indication of multicollinearity issues (all bivariate correlation coefficients smaller than 0.6). In terms of potentially influential cases, no cases had a value of Cook’s distance larger than 1, calculated average leverage larger than three times the average value nor close to 1, nor absolute value of DFBeta (a statistic that indicates the effect of deleting an observation on the regression coefficient) larger than 1. Based on the standardized residuals, less than 5% of cases had absolute values above 2 (3.6%) and less than 1% of cases above 2.5 (0.2%). Nevertheless, there were nine cases with a value of standardized residuals above 3, which could be potential outliers. The logistic regression model was tested in the sample with these nine potential outliers removed (*n* = 1580). Compared to the model based on the original sample (*n* = 1589), the goodness-of-fit improved from 28.6% to 30.8% based on Nagelkerke R Square and is thus reported as the final model. 

Differences in the perceived amount of protein in the current diet, intention to keep consuming the same amount of protein in the diet, and likelihood to increase the amount of protein in their daily diet if they were told to do so by a specific source between those with lower and higher protein knowledge were tested with chi-square tests. Differences in the frequency of used media and used information sources for obtaining information on new food products between those with lower and higher protein knowledge were tested with the Mann–Whitney U test.

## 3. Results

Table 1 provides an overview of the sample characteristics. The sample was 49.6% female and 87.0% able to walk without difficulties. The mean age was 69.8 years (SD = 4.1) and mean BMI was 27.6 kg/m^2^ (SD = 6.4). 

**Figure 1:**
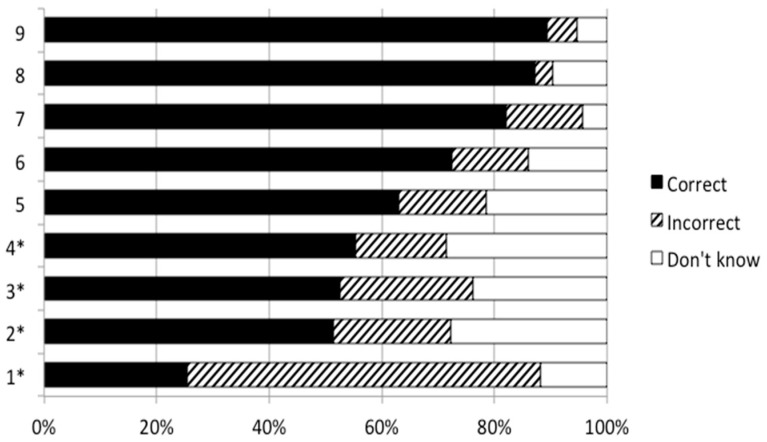
Percentage of correct, incorrect and don’t know answers in the objective protein knowledge test about dietary protein in 1180 participants who indicated to know what dietary protein is. * False statements (i.e., “No” was the correct answer to these statements). The full text of the statements is shown in Table 2.

Of the sample, 35.3% indicated not to know what dietary protein is. Participants scored best on the true statement "You need protein in the diet for repairing bones and muscles" (10.7% answered incorrect or don’t know), and worst on the false statement "One meal per day with a good protein source is sufficient" (74.6% answered incorrect or don’t know) (Figure 1). In general, statements related to the relationship between protein and health were scored best, while statements related to the protein recommendations were scored worst. Median protein knowledge score of the 1180 participants receiving all nine statements was 5.0. Lower protein knowledge was observed in 902 (49.4%) participants of the total study sample. 

Table 2. shows the responses to the nine protein knowledge statements stratified for those with a lower and a higher protein knowledge score. Participants with a lower protein knowledge score scored consistently lower on all nine statements (*p* < 0.001).

To identify potential determinants of lower protein knowledge in older adults, a multiple regression analysis was performed using data from 1580 participants (Table 3). Males (OR 1.57) and those unable to walk for 5 min (2.66), not always making own food decision (1.36) and having a lower income (1.44) were more likely to have lower protein knowledge. Compared to Finland, all other countries in the study were more likely to have lower protein knowledge (3.78–27.24), with older adults from Spain being at the highest risk. Variables entered but not retained in the model, included: age (continuous); education (dichotomous); living with assistance (dichotomous); living alone (dichotomous); main household grocery shopper (dichotomous); ability to prepare own warm meals (dichotomous); perceived financial situation (dummy); food expenditure at home (dichotomous); food expenditure out of home (dichotomous); diet status (dichotomous); and BMI (continuous).

The majority of participants (67.4%) considered the amount of protein in their daily diet to be "just about right". (Slightly) too much was reported by 7.0% of the total sample, (slightly) too little by 15.1%, and don’t know by 10.5%. When stratified according to lower and higher protein knowledge (Table 4), those with lower protein knowledge were more likely to report "don’t know", and less likely to report "(slightly) too much" or "(slightly) too little" compared to those with higher protein knowledge (*p* < 0.001). 

The majority of participants reported to have the intention to keep consuming the same amount of protein in the diet (66.4%), while 10.0% reported the intention to increase the amount, and 4.9% to decrease the amount. Those with lower protein knowledge less often reported the intention to increase or remain stable, and more often reported not to know compared to those with higher protein knowledge (Table 4). 

**Figure 2:**
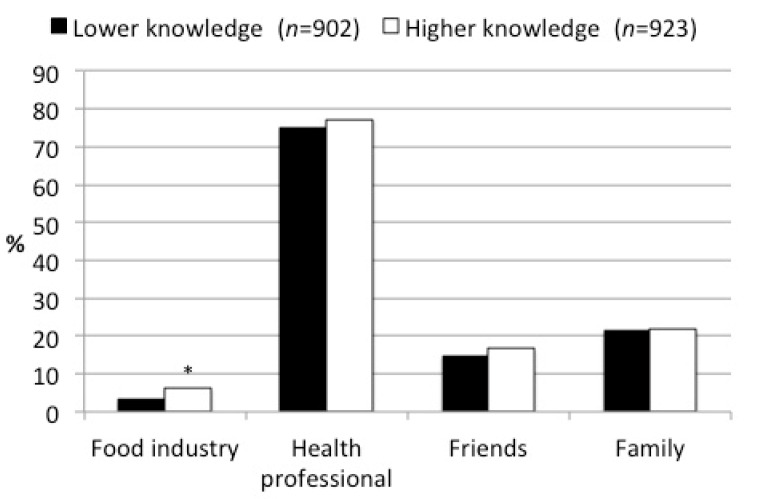
Proportions of the participants in the two protein knowledge level groups who would increase the amount of protein in current diet if told to do so by the information source (*n* = 1825). * Significant difference between the proportions of the two knowledge groups based on chi-square test (*p*-value < 0.01). The percentages refer to the responses of "yes" as opposed to "no" or “don’t know”.

Participants were more likely to increase the amount of protein in their daily diet if they were told by a health professional (i.e., physician or dietician) (76.2%) as compared to being told by the food industry (4.8%), family (21.7%) or friends (15.7%). There were no differences between those with lower or higher protein knowledge, with the exception for food industry (Figure 2). Participants with lower protein knowledge were less likely to increase the amount of protein in their daily diet when told by the food industry (3.2%) as compared to those with higher protein knowledge (6.4%, *p* < 0.001).

**Figure 3:**
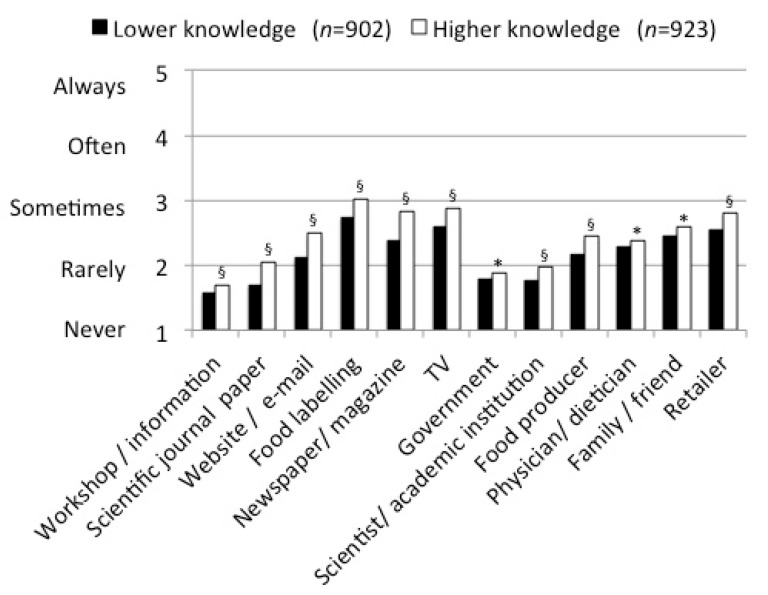
Frequency of used media sources and used information sources for obtaining information about new food products for the two protein knowledge groups separately (*n* = 1825). * Significant difference between the two knowledge groups based on the Mann–Whitney U test at the <0.05 level, § at the <0.001 level.

The average frequency of using media sources and information sources for obtaining information about new food products for all older adults and stratified by lower and higher protein knowledge is shown in Figure 3. Participants with lower protein knowledge consistently used all media sources and information sources less frequently compared to those with higher protein knowledge. The five most frequently used sources in both groups were: food labelling, television, newspaper/magazine, retailer and family/friend. 

## 4. Discussion

The results of this large-scale survey among community-dwelling older adults from five European countries suggest that protein knowledge has substantial room to improve, and, in particular, knowledge on the optimal intake of protein. Risk groups with lower protein knowledge are older adult males, those unable to walk for 5 min, those not always making their own food decision and those having a lower income. Furthermore, large differences in protein knowledge between countries were observed. Older adults with lower protein knowledge were more uncertain whether the amount of protein in their daily diet is about right and whether they should intend to change or keep consuming the same amount of protein in the diet. Both the lower and higher knowledge group were most likely to increase the amount of protein in the current diet if told to do so by a health professional. Older adults with lower protein knowledge less frequently used media sources and information sources about new food products than those with higher protein knowledge; however, the type of sources they used most frequently was similar to those with higher protein knowledge. 

About one-third of the sample indicated not to know what dietary protein was. In addition, among those who reported that they knew, more than one-fifth had a poor score on the protein knowledge test. These results illustrate that knowledge on dietary protein is poor in many older adults. Regarding the three components of protein knowledge tested in our study, our data showed that knowledge on the relation between protein and health was highest, and that knowledge on the recommended intake of protein was lowest among older adults. This ranking remained similar when stratified by lower and higher protein knowledge. A previous study conducted in a convenience sample of 190 community-dwelling older adults living in the United States also showed that knowledge on the link between protein and health is higher as compared to knowledge on the recommended protein intake for older adults [14]. These results suggest that future communication strategies should predominantly focus on the recommended intake of protein for older adults, but also on the protein content of food. Previous research has shown that higher nutritional knowledge on all three components is associated with healthier food purchasing behaviours [11]. The high level of knowledge on the relation between protein and health, as observed in our study sample, emerges as a good starting point for future improvements. 

Previous studies conducted in community-dwelling adults aged 65 and higher have shown that greater protein knowledge is linked to higher protein intake and that an increase in protein knowledge was paralleled by an increase in protein intake [6,7]. The poor protein knowledge observed in our study could explain why many older adults have protein intakes below the current recommendation [3]. However, it should be acknowledged that other factors determine the consumption of protein-rich foods, such as liking and convenience [6,15]. Furthermore, motivation has been shown to be a strong determinant of dietary behaviour [16]. Consumers need to experience a need for health-related information, which is driven by an interest in healthy eating, to become motivated. Therefore, communication strategies should not merely focus on providing protein knowledge, but should also try to increase consumers’ motivation to increase their protein intake, in order for the provided knowledge to become effective. 

As protein plays an important role in the environmental impact of the diet, strategies to increase protein knowledge should also include information on alternative and more sustainable protein sources. Currently, red meat and poultry are preferred above plant-based protein-enriched products among the European older adults [17]. While plant-based protein is a well-accepted protein source for older adults [10], other alternatives such as single-cell protein, insect-based protein and cultured meat protein were less accepted, especially in older adults with higher levels of food fussiness, and those with less green eating behaviour and those with lower educational attainment. 

Our study shows that males, those unable to walk for 5 min, those not always making their own food decision and those having a lower income were more likely to have a lower protein knowledge. Communication strategies should be designed to target these specific subgroups and be developed in such a way that the information can be processed by these receiving subgroups [8]. We also observed large regional differences in protein knowledge, with the highest knowledge in Finland. Finland is the only country of all the countries included in our survey where the recommended daily allowance for protein is set at 1.2 g per kg body weight per day, which is higher than the recommendation of 0.8 g per kg body weight per day for the other countries. The higher recommendation in Finland may have caused increased attention for sufficient protein consumption in national and regional health campaigns, potentially explaining the higher protein knowledge in Finland. 

A strength of this study is the use of a large dataset obtained through a survey across five countries in Europe. In addition, this study is one of the first attempts to investigate the protein knowledge of older adults, as well as the determinants that are associated with lower protein knowledge in older adults. The potential limitations of the study should also be acknowledged. Firstly, we observed that that the four false statements used in the objective knowledge test had a lower percentage of correct answers, as compared to the five true statements, despite randomising the presentation order of these statements. While our study shows that knowledge on the recommended intake of protein is lower than knowledge on the relation between protein and health, we cannot exclude that this difference was partly caused by the fact that the statements regarding the recommended intake of protein were included as false statements. However, a previous study conducted in independently living older adults also showed that older adults were most knowledgeable on the role of proteins in health but were generally more uncertain whether their protein intake was sufficient [18]. The objective protein knowledge statements were developed based on insights of nutrition and/or geriatric experts in the research consortium. To our knowledge, most validated instruments that measure general nutrition knowledge cover multiple nutrients but contain few questions specifically related to protein. In order to account for the different aspects of protein knowledge, namely "relation protein and health"; "protein content of foods"; "recommended intake of protein", the objective knowledge statements were newly developed for the purpose of this study without using a validated instrument. A second possible limitation relates to the study’s two-step procedure to assess protein knowledge. A dichotomous measure of perceived knowledge was used to filter participants who believed that they knew what protein is, who were then directed to a set of objective knowledge statements. The rationale for this approach was to avoid guessing on the objective knowledge statements among participants who claimed not to know what protein is. Meanwhile, this approach prevented us from investigating the eventual differential impact of subjective (or perceived) versus objective (or real) knowledge. Consumer studies point out that subjective knowledge might be a stronger determinant of food consumption compared to objective knowledge [19,20]. These studies conclude that apart from providing factual information to build objective knowledge, it is also important to foster people’s belief of being knowledgeable in order for communication to be effective. Future studies might, therefore, also specifically investigate subjective dietary protein knowledge of older adults and its link with protein intake. A final limitation is that the frequency of used information sources referred to new food products in general and not specifically to protein-rich or protein-enriched food products. Therefore, we cannot exclude that different information sources are used by older adults to obtain information, specifically that on protein-(en)rich(ed) food products.

In conclusion, the results of this large-scale survey across Europe suggest that knowledge about dietary protein is rather low in half of the older population. Specific subgroups of older adults with lower knowledge were identified and should be reached by targeted communication strategies. Food labelling, television, newspaper/magazine, retailer and family/friend are the most frequently used sources of information to obtain knowledge on new food products in older adults with both lower and higher dietary protein knowledge, and these sources could be used to increase protein knowledge in older persons. Finally, health professionals, such as physicians and dieticians, can play an important role in increasing the amount of protein in the diets of older adults, as these sources emerged as potentially the most influential.

## Figures and Tables

**Table 1:** Sample characteristics (% of respondents, *n* = 1825).

		Sample (%)
Gender	Male	50.4
	Female	49.6
Age group	<70 years	55.9
	70 years or above	44.1
Country	Finland	20.0
	Poland	20.0
	Spain	19.9
	The Netherlands	20.1
	United Kingdom	20.0
Education level	Below tertiary level	59.6
	Tertiary level or above	40.4
Living with assistance	Yes	11.3
	No	88.7
Household size	Single-person	30.6
	Multi-person	69.4
Main household grocery shopper	Yes	70.3
	No or shared responsibility	29.7
Making own food decision	Always	69.3
	Sometimes or never (someone else decides)	30.7
Ability to prepare own warm meals	Able to prepare without difficulties	89.6
	Able to prepare but with difficulties	4.9
	Unable to or never prepare	5.5
Ability to walk (or move own wheelchair) for 5 min without resting (*n* = 1822)	Able to walk without difficulties	87.0
	Able to walk but with difficulties	9.3
	Unable to walk	3.7
Perceived financial situation (*n* = 1791)	Manage quite or very well	45.3
	Get by alright	38.3
	Have some or severe difficulties	16.4
Monthly net household income (*n* = 1589)	<€1500	59.1
	€1500 or above	40.9
Food expenditure at home (*n* = 1297)	<€60	42.8
	€60 or above	57.2
Food expenditure out of home (*n* = 1269)	<€30	72.1
	€30 or above	27.9
Following a diet *	No	89.0
	Yes	11.0
Low BMI (<70 years, *n* = 917)	<20 kg/m^2^	2.2
Low BMI (70 years or above, *n* = 744)	<22 kg/m^2^	9.7

* Such as a vegetarian diet, vegan diet or any other diet.

**Table 2:** Number (*n*) and percentage of participants (%) with a correct answer for each of the nine protein knowledge statements by the two knowledge level groups (*n* = 1180).

	Lower Knowledge Group ^#^ (*n* = 257)		Higher Knowledge Group (*n* = 923)		*p*-Value
Relation protein and health	*n*	%	*n*	%	
1 “You need protein in the diet for repairing bones and muscles”	167	65.0	887	96.1	<0.001
2 “You need protein in the diet for building body cells”	163	63.4	865	93.7	<0.001
3 “You need protein in the diet for energy”	167	65.0	801	86.8	<0.001
4 “You will experience loss in muscle mass if you do not consume enough protein”	79	30.7	777	84.2	<0.001
Protein content of foods					
5 “Cooked lean beef has more protein than the same amount of cooked tomato”	80	31.1	662	71.7	<0.001
6 “Whole milk (100 mL) has more protein than cheese (100 g)” *	68	26.5	584	63.3	< 0.001
Recommended intake of protein					
7 “The human body is good at storing protein to use it later, it is thus not necessary to consume a steady amount of protein every day” *	47	18.3	574	62.2	<0.001
8 “Health experts recommend people of my age to consume less protein” *	47	18.3	558	60.5	<0.001
9 “One meal per day with a good protein source is sufficient” *	17	6.6	283	30.7	<0.001

^#^ Excluding participants who indicated that they do not know what dietary protein was. * False statements (i.e., “No” is the correct answer to these statements). *p*-values are based on chi-square tests.

**Table 3:** Determinants of lower protein knowledge in community-dwelling adults aged 65 years and above living in five European countries (*n* = 1580).

	Odds Ratio	Bootstrapped 95% Confidence Interval	
		Lower	Upper
Country (ref: Finland)			
Spain	27.24 **	17.33	42.81
United Kingdom	15.42 **	9.97	23.85
The Netherlands	7.64 **	4.97	11.73
Poland	3.78 **	2.34	6.10
Gender (ref: Female)			
Male	1.57 *	1.21	2.04
Ability to walk (ref: able to walk without difficulties)			
Able to walk with difficulties	1.19	0.81	1.75
Unable to walk	2.67 *	1.36	5.19
Household income (ref: 1500 EUR and above)			
Less than 1500 EUR per month	1.44 *	1.08	1.91
Making own food decision (ref: always)			
Sometimes or never	1.36 *	1.03	1.80

** *p* ≤ 0.001; * *p* < 0.05 based on robust method with 1000 bootstrap samples. Bootstrapped 95% confidence interval based on bias-corrected and accelerated method. Model goodness-of-fit: Nagelkerke R Square = 30.8%.

**Table 4:** Perceived amount of protein in the current diet and intention to change the amount of protein in the current diet by the two protein knowledge level groups (*n* = 1825).

	Lower Protein Knowledge (*n* = 902)	Higher Protein Knowledge (*n* = 923)
**Perceived amount of protein in the current diet**		
(Slightly) too much	5.3	8.6 *
Just about right	65.6	69.2
(Slightly) too little	12.8	17.3 *
I don’t know	16.3	4.9 *
**Intention to change the amount of protein in the current diet**		
Yes, increase the amount	6.4	13.4 *
No, remain the same	4.8	5.1 *
Yes, decrease the amount	63.0	69.7
I don’t know	25.8	11.8 *

* significant difference between the proportions of the two knowledge groups based on chi-square test (*p*-value < 0.001).

## Data Availability

The data presented in this study are available on request from the last author (Email: wim.verbeke@ugent.be).
